# Alkaline Chemical Polishing Combined with Silane Electrodeposition for Improving Etched Tunnel Distribution in Aluminum Foil

**DOI:** 10.3390/ma19101922

**Published:** 2026-05-07

**Authors:** Jinlong Wu, Huwei Tao, Wenfeng Yang, Bowei Zhang, Junsheng Wu

**Affiliations:** 1 Institute of Advanced Materials and Technology, University of Science and Technology Beijing, Beijing 100083, China; 2 Xinjiang Joinworld Company Limited, Urumuqi 830000, China

**Keywords:** aluminum foil, alkaline chemical polish, silane electrodeposition, etched tunnels

## Abstract

A combined pretreatment strategy involving alkaline chemical polishing and silane electrodeposition was proposed for regulating the surface state of aluminum foil and the formation of etched tunnels during DC tunnel etching. Electrochemical measurements and morphological characterization were used to evaluate the effects of this pretreatment on surface electrochemical activity and etched tunnel structure. The results showed that appropriate alkaline chemical polishing facilitated the removal of rolling-induced surface relief, improved the uniformity of surface electrochemical activity, and favored the uniform deposition of the silane film. In contrast, excessive polishing generated surface pits during the polishing process, and these preformed pits subsequently promoted tunnel merging during DC tunnel etching. Under the optimal processing conditions, the combined pretreatment significantly improved the distribution uniformity and dimensional consistency of etched tunnels and suppressed tunnel merging. Under the present testing conditions, the specific capacitance increased from 0.386 to 0.509 μF cm^−2^, corresponding to an improvement of approximately 31.9%. This work provides an effective approach for optimizing etched tunnel structure and improving the capacitance-related performance of aluminum capacitor foil.

## 1. Introduction

Aluminum electrolytic capacitors have gained a significant share of the capacitor market due to their low cost, high capacitance, and high operating electric field strength, and are widely used in automotive electronics, aerospace, computers, and other fields [1,2,3,4]. With the continuous advancement of science and technology, capacitors are developing toward miniaturization and higher specific capacitance, which imposes more stringent requirements on their key manufacturing technologies [5,6].

Aluminum electrolytic capacitors mainly consist of anode foil, cathode foil, and electrolyte, among which the anode foil is the key component governing capacitor performance [6,7,8,9]. After decades of development, electrochemical DC etching of anode aluminum foil in acidic chloride-containing solutions at elevated temperatures has become the main industrial strategy for enhancing the specific capacitance of aluminum electrolytic capacitors, owing to its ability to generate high-density etched tunnels and thus enlarge the specific surface area [10,11,12]. Therefore, determining how to obtain etched tunnels with high density and maximum surface area utilization remains a major research focus in this field. Previous studies have extensively examined the effects of crystallographic texture, trace elements, grain size, and etching parameters, including electrolyte composition, current density, and temperature, on anode aluminum foil [9,13,14,15,16,17]. Recent studies have shown that the surface state of blank aluminum foil, which serves as the starting material for aluminum electrolytic capacitor anode foil, has a pronounced influence on the distribution of etched tunnels during the etching process. During foil manufacture, surface defects introduced by rolling, such as rolling marks, oil contamination, and cracks in the oxide film, can act as preferential sites for initial pit nucleation during DC etching, thereby leading to tunnel merging, reduced surface utilization, and consequently inferior capacitor performance. Although cleaning steps are usually applied before etching in industrial production to remove oil contamination and other impurities, the distribution of etched tunnels remains unsatisfactory [18,19,20].

In recent years, improving the surface state of blank aluminum foil through pretreatment prior to DC etching has become an emerging research focus in the field. Peng et al. [21] induced more uniform tunnel corrosion by depositing Zn nuclei on the aluminum foil surface to form Zn–Al micro-galvanic cells, thereby improving the specific capacitance. Liu et al. [22] deposited Cu inside the primary etched tunnels, which promoted the formation of a large number of lateral branch tunnels and further enhanced the capacitive performance of the aluminum foil. Hwa S. P. et al. [23] employed a polyimide layer to construct an ordered mask on the aluminum foil surface, thereby guiding tunnel initiation at the exposed sites of the mask and improving the ordering of tunnel distribution. Feng et al. [24] used 3-mercaptopropyltriethoxysilane to form a porous thin film on the aluminum foil surface, which regulated the growth and distribution of the etched tunnel and improved their size consistency. These studies demonstrate that surface pretreatment can effectively regulate tunnel initiation and growth behavior, and thus provides a promising strategy for optimizing the etched tunnel structure of capacitor foil.

However, the DC etching process is essentially governed by the surface electrochemical activity of the aluminum foil. Existing studies still lack an industrially applicable pretreatment method capable of effectively homogenizing the surface activity distribution. In practical industrial production, the distribution of etched tunnels is still far from the optimal tunnel structure, and the specific capacitance remains significantly below the theoretical value. Therefore, the development of a more effective combined pretreatment strategy is of considerable significance for further regulating etched tunnel distribution during electrochemical DC etching, improving the specific capacitance of aluminum electrolytic capacitor foil, and promoting the advancement of the aluminum electrolytic capacitor industry.

In this work, the effectiveness of alkaline chemical polishing under different processing parameters in removing rolling marks from the aluminum foil surface and its influence on surface electrochemical activity were investigated. The promoting effect of alkaline chemical polishing on silane electrodeposition was also examined. Based on the optimal polishing conditions, the effects of the combined treatment of alkaline chemical polishing and silane electrodeposition on etched tunnel distribution and structural parameters during electrochemical DC etching were further studied, and the most effective combined pretreatment strategy was identified.

## 2. Materials and Methods

### 2.1. Preparation of Materials

The aluminum foil used in this study was a high-purity foil for electrolytic capacitors, with a purity of 99.99%, a cube texture (100) fraction higher than 95%, and a thickness of 140 μm, and was manufactured by Joinworld (Urumuqi, China). Before the experiments, the aluminum foil was ultrasonically cleaned in acetone for 10 min to remove organic contaminants from the surface, then rinsed with deionized water and dried in air for subsequent use.

### 2.2. Alkaline Chemical Polishing

To remove the native oxide layer and rolling marks, thereby improving the surface activity for the subsequent electrochemical (DC) tunnel etching, the cleaned aluminum foil (from Section 2.1) was treated by immersion in an aqueous solution of 1–5 wt% NaOH (Aladdin Reagent (Shanghai) Co., Ltd., Shanghai, China) and 0.5 wt% sodium tartrate for 1–5 min, with the bath temperature varied between 30 and 60 °C, followed by thorough rinsing with deionized water and drying under an air stream.

### 2.3. Silane Electrodeposition Treatment

BTSE-modified solutions were prepared by first adjusting the pH of an ethanol/deionized water mixture (85:15, *v*/*v*) to 4.0–4.5 with acetic acid (Aladdin Reagent (Shanghai) Co., Ltd., Shanghai, China). Subsequently, 1,2-bis(triethoxysilyl)ethane (BTSE, Aladdin Reagent (Shanghai) Co., Ltd., Shanghai, China) was added at different concentrations (1, 3, 5, and 7 vol%), and the mixtures were stirred at room temperature for 48 h to ensure sufficient hydrolysis [25,26,27]. Electrochemical deposition was carried out in a conventional three-electrode system, in which the aluminum foil pretreated by alkaline cleaning, as described in Section 2.2, served as the working electrode, a saturated calomel electrode (SCE) was used as the reference electrode, and a platinum sheet acted as the auxiliary electrode. The prepared BTSE solution was used as the electrodeposition electrolyte, and a constant potential of −0.8 V vs. SCE was applied to the sample electrode for different deposition times (1, 5, 10, and 20 min). After deposition, the samples were removed and thermally cured in an oven at 110 °C for different durations (5, 15, and 30 min).

### 2.4. DC Tunnel Etching

DC tunnel etching was conducted in a mixed acid electrolyte containing 0.6 M HCl and 3.5 M H_2_SO_4_ at 75 °C with continuous stirring. The etching was performed at a constant current density of 500 mA cm^−2^ for 60 s, using aluminum foil (exposed area: 45 cm^2^) as the anode and a graphite plate (exposed area: 45 cm^2^) as the cathode.

### 2.5. Surface Characterization

Electrochemical measurements were conducted in a conventional three-electrode cell using an electrochemical workstation (Metrohm Autolab, Utrecht, The Netherlands). The working electrode was aluminum foil with an exposed area of 1 cm^2^, while the remaining surface was sealed with 704 silicone rubber. An Ag/AgCl electrode and a platinum sheet were used as the reference and auxiliary electrodes, respectively. The electrolyte was the same as that used for DC tunnel etching, i.e., 0.6 M HCl + 3.5 M H_2_SO_4_. Electrochemical impedance spectroscopy (EIS) measurements were performed over the frequency range from 1 to 10^5^ Hz at an applied potential of −0.669 V vs. SCE. Polarization curves were recorded at a scan rate of 10 mV·s^−1^.

To facilitate observation of the etched tunnels, the etched aluminum foil was electropolished in a solution containing 20 vol% HClO_4_ and 80 vol% ethanol at 0 °C under 20 V for 30 s. The tunnel morphology was then examined by scanning electron microscopy (SEM, FEI Quanta 250). Structural parameters of the etched tunnels, including the average diameter, density, and size distribution, were statistically analyzed using Image-Pro Plus 6.0.0.260 (IPP) software. Oxide replicas of the etched tunnels were prepared by anodizing the samples at 30 V for 20 min in ammonium adipate solution (150 g·L^−1^, AR, Aladdin Reagent (Shanghai) Co., Ltd., Shanghai, China) at 85 °C. The replicas were subsequently immersed in 10 wt% I_2_-methanol solution to dissolve the remaining Al substrate and expose the inner surface. After Au sputtering, the oxide replicas were examined by SEM (FEI Quanta 250, FEI Company, Hillsboro, OR, USA) to characterize the cross-sectional morphology of the etched tunnels. To compare the specific capacitance of the etched aluminum foils, the samples were first anodized at 520 V for 20 min in a boric acid solution (100 g/L, AR, Aladdin Reagent (Shanghai) Co., Ltd., Shanghai, China) at 85 °C using stainless steel as the counter electrode. The specific capacitance was then measured in an ammonium pentaborate solution (80 g/L) at 30 °C using an LCR meter (TH2830, Tonghui Electronics Co., Ltd., Changzhou, China) at 120 Hz with a signal level of 250 mV. During the capacitance measurement, a cathode foil with a capacitance exceeding 40,000 μF was used as the counter electrode.

## 3. Results and Discussion

### 3.1. Effect of Alkaline Chemical Polishing Parameters on Aluminum Foil Surface State and Etched Tunnel Structure

**Figure 1:**
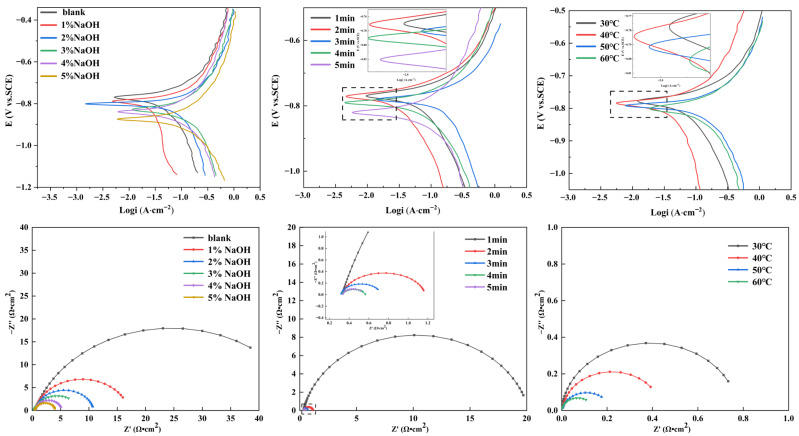
Electrochemical test results of Al foils treated under different polishing conditions.

As shown in Figure 1, the alkaline-polished aluminum foils exhibited a smaller capacitive arc in the Nyquist plots and a negative shift in the polarization curves compared with the blank foil, indicating enhanced electrochemical activity in the etching solution. This behavior is attributed to the dissolution of the native passive oxide film in the alkaline medium, which exposes the fresh aluminum substrate and increases the surface reactivity. As the NaOH concentration, immersion time, and treatment temperature increased, the capacitive arc decreased further and the polarization curves shifted to more negative potentials, suggesting lower charge-transfer resistance and higher corrosion susceptibility. This results from the progressive dissolution of the aluminum substrate in the alkaline solution, which creates more active sites and local surface defects for subsequent etching. Therefore, the alkaline polishing parameters should be carefully controlled to achieve an appropriate balance between passive film removal and surface activity.

**Figure 2:**
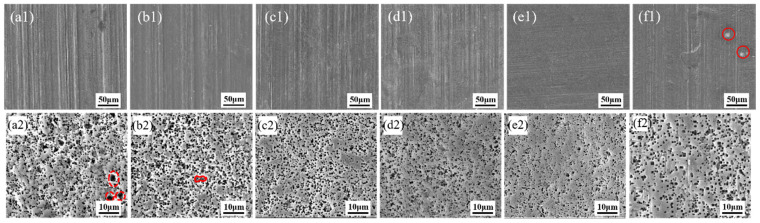
Surface morphologies and etched tunnel morphologies of aluminum foils after alkaline chemical polishing in solutions containing (**a1**,**a2**) 0 wt% NaOH (blank), (**b1**,**b2**) 1 wt% NaOH, (**c1**,**c2**) 2 wt% NaOH, (**d1**,**d2**) 3 wt% NaOH, (**e1**,**e2**) 4 wt% NaOH, and (**f1**,**f2**) 5 wt% NaOH.

Figure 2 presents the surface rolling marks and etched tunnel morphologies of aluminum foils subjected to alkaline polishing in NaOH solutions with different concentrations. As shown in Figure 2(a1), the blank aluminum foil exhibited numerous parallel rolling marks on the surface. Owing to impurity/dislocation enrichment and the associated higher local lattice energy, these rolling marks can act as highly active regions during electrochemical etching [14,18], thereby promoting the preferential initiation of etched tunnels and resulting in a large number of merged tunnels, as marked in red in Figure 2(a2). Increasing the NaOH concentration improved the removal of rolling marks, thereby reducing the localized clustering of etched tunnels along these regions and enhancing the uniformity of etched tunnel distribution, as shown in Figure 2(b1,b2,c1,c2,d1,d2,e1,e2). However, when the NaOH concentration reached 5 wt%, a large number of merged tunnels reappeared. This was attributed to the formation of pits on the aluminum foil surface at an excessively high alkali concentration, as shown in the red-marked region of Figure 2(f1).

**Figure 3:**
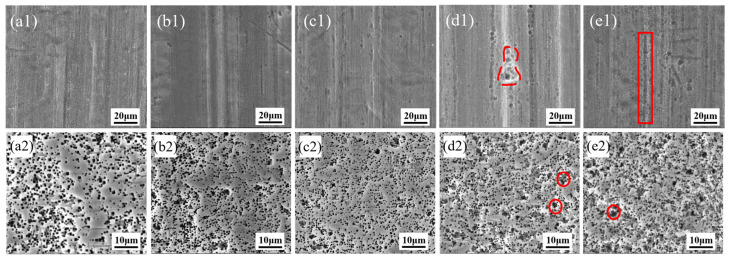
Surface morphologies and etched tunnel morphologies of aluminum foils after alkaline chemical polishing for (**a1**,**a2**) 1 min, (**b1**,**b2**) 2 min, (**c1**,**c2**) 3 min, (**d1**,**d2**) 4 min, and (**e1**,**e2**) 5 min.

Figure 3 presents the surface morphologies and etched tunnel morphologies of aluminum foils after different immersion times. As shown in Figure 3(a1,a2,b1,b2), short immersion times were insufficient in effectively eliminating the rolling marks on the aluminum foil surface, resulting in relatively high surface roughness and non-uniform surface activity. As a result, the distribution uniformity of etched tunnels was not significantly improved, and a large number of merged tunnels remained. At an immersion time of 3 min, a small number of circular pits appeared on the aluminum foil surface (Figure 3(c1)) and were preferentially distributed along the rolling direction. With further increasing immersion time, the pits increased in both number and size, and gradually coalesced into larger corrosion pits, as indicated by the red dashed and solid marked regions in Figure 3(d1,e1). This was caused by corrosion of the aluminum foil surface by the alkaline solution at longer immersion times. During subsequent electrochemical etching, such pits strongly induced the localized clustering of etched tunnels, resulting in the formation of more merged tunnels, as shown by the red-circled regions in Figure 3(d2,e2).

**Figure 4:**
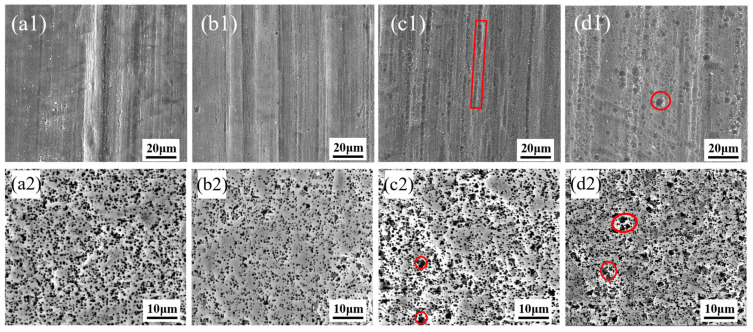
Surface morphologies and etched tunnel morphologies of aluminum foils after alkaline chemical polishing at (**a1**,**a2**) 30 °C, (**b1**,**b2**) 40 °C, (**c1**,**c2**) 50 °C, and (**d1**,**d2**) 60 °C.

Figure 4 presents the surface morphologies and etched tunnel morphologies of aluminum foils after alkaline polishing at different immersion temperatures. The effect of temperature was consistent with that of NaOH concentration and immersion time. When the immersion temperature was excessively high, localized pits appeared on the aluminum foil surface, as indicated by the red-marked regions in Figure 4(c1,d1), and the corresponding etched tunnels exhibited pronounced merging, as shown by the red-circled regions in Figure 4(c2,d2). This is attributed to the chemical reaction between Al and the NaOH solution during alkaline polishing, as given in Equation (1). The dissolution of the native oxide film exposes the fresh Al substrate, which then reacts with the alkaline solution to form aluminate species and H_2_. Because the reaction involves a positive entropy contribution associated with hydrogen evolution, increasing temperature enhances the thermodynamic driving force of the reaction. Meanwhile, the interfacial reaction kinetics are also accelerated, thereby intensifying the attack of the alkaline solution on the Al substrate. These pits act as preferential initiation sites for subsequent tunnel etching, leading to local tunnel clustering and merged tunnels.

(1) $$2Al+2NaOH+6H_{2}O\to 2Na\left[Al{\left(OH\right)}_{4}\right]+3H_{2}↑$$

**Figure 5:**
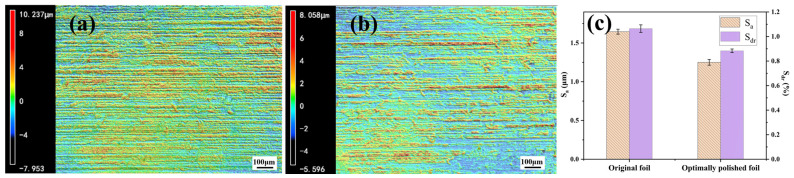
Surface topographies and roughness parameters of the original Al foil and the optimally polished Al foil: (**a**) original Al foil; (**b**) optimally polished Al foil; (**c**) comparison of the surface roughness parameters S_a_ and S_dr_. Error bars represent mean ± SD based on measurements obtained from three different regions of the same sample (*n* = 3).

As shown in Figure 5, the original aluminum foil exhibited obvious stripe-like surface relief along the rolling direction and a relatively high surface roughness. After alkaline chemical polishing under the optimal conditions, the surface became flatter, and both S_a_ and S_dr_ decreased significantly. Moreover, the relatively small error bars indicate limited variation in roughness among different regions of the same sample. These results suggest that appropriate alkaline chemical polishing can effectively weaken the surface non-uniformity caused by rolling marks and reduce the surface roughness of the aluminum foil.

**Figure 6:**
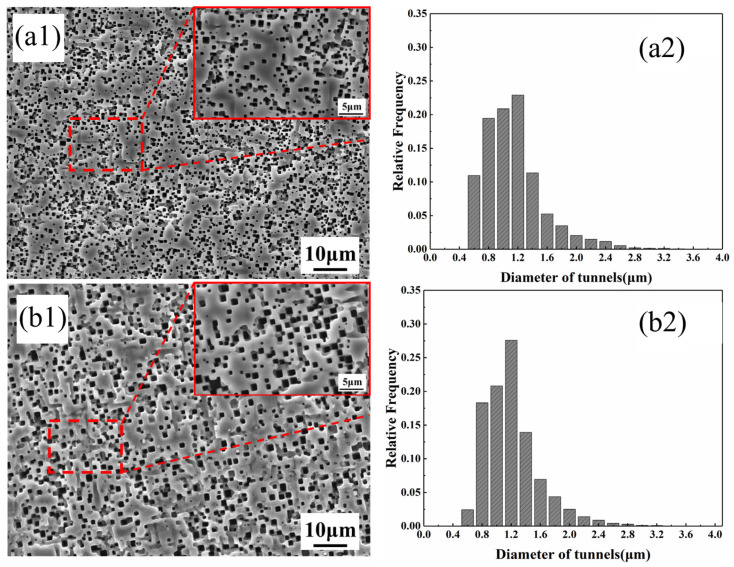
Etched tunnel morphologies and tunnel diameter distributions of aluminum foils after (**a1**,**a2**) silane treatment and (**b1**,**b2**) combined pretreatment of alkaline chemical polishing and silane deposition.

Figure 6 presents the etched tunnel morphologies and tunnel diameter distributions of aluminum foils subjected to the combined treatment of alkaline chemical polishing and silane modification. After alkaline chemical polishing pretreatment, the silane-treated aluminum foil exhibited a more uniform etched tunnel distribution, fewer merged tunnels, and a narrower tunnel diameter distribution, although the tunnel density decreased slightly. This is attributed to the reduced surface roughness and more homogeneous surface activity after polishing, which facilitate the uniform deposition of the silane film on the aluminum foil and thereby promote a more uniform current distribution during DC tunnel etching. In addition, alkaline chemical polishing generates surface hydroxyl groups on the aluminum foil, strengthening the interfacial bonding between the silane film and the aluminum substrate and thus enhancing the corrosion inhibition effect of the silane film [28,29].

### 3.2. Effect of Silane Electrodeposition Parameters on Etched Tunnel Structure Under Combined Pretreatment

To determine the optimal combined pretreatment of alkaline chemical polishing and silane deposition, the polished aluminum foils were subjected to silane electrodeposition at different silane concentrations, deposition times, and curing times. The treated aluminum foils were then subjected to electrochemical DC etching. The optimal conditions were identified by comparing the morphologies and structural parameters of the etched tunnels.

**Figure 7:**
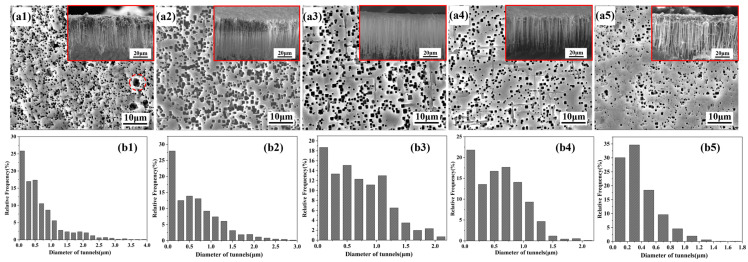
Etched tunnel morphologies and tunnel diameter distributions of aluminum foils at different silane concentrations: (**a1**,**b1**) blank, (**a2**,**b2**) 1 vol% silane, (**a3**,**b3**) 3 vol% silane, (**a4**,**b4**) 5 vol% silane, and (**a5**,**b5**) 7 vol% silane.

As shown in Figure 7, the uniformity of etched tunnel distribution on the aluminum foil improved with increasing silane concentration. This is attributed to the enhanced uniformity of the silane film formed on the aluminum foil surface at higher silane concentrations, which promoted a more homogeneous distribution of corrosion current during electrochemical DC etching and thereby improved the distribution of etched tunnels. The red dashed circle in Figure 7(a1) marks a locally merged tunnel region formed at low silane concentration. As indicated by the cross-sectional morphologies marked in red, the length uniformity of the etched tunnels gradually increased with increasing silane concentration and then remained nearly unchanged. This is because the enhanced corrosion inhibition effect of the silane film made the growth duration of the etched tunnels more consistent, thereby improving their length uniformity. Meanwhile, the cross-sectional morphologies also revealed a clear decreasing trend in etched tunnel density. Figure 7(b1–b5) shows the tunnel diameter distribution, from which it can be seen that the maximum tunnel diameter decreased progressively with increasing silane concentration. This is consistent with the surface morphology evolution shown in Figure 7(a1–a5) and is attributed to the enhanced corrosion inhibition ability of the silane film.

**Figure 8:**
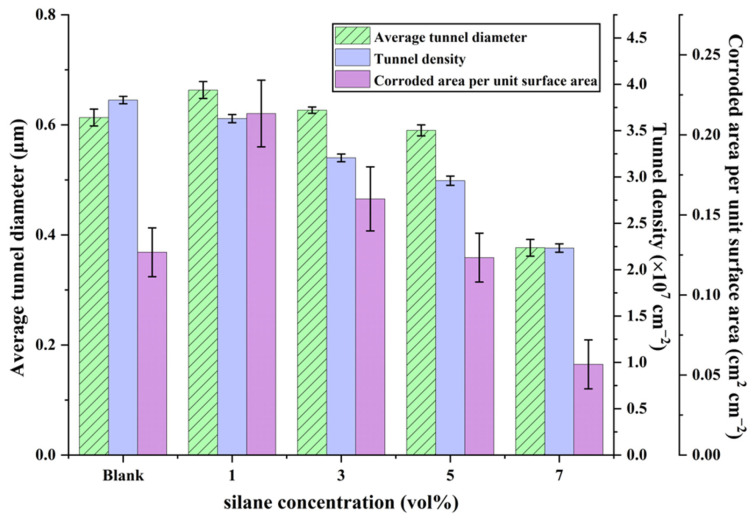
Statistical analysis of the structural parameters of the etched tunnels shown in Figure 7. Error bars represent mean ± SD based on measurements obtained from three SEM images taken from different regions of the same sample (*n* = 3).

Figure 8 presents the statistical results of the structural parameters of the etched tunnels shown in Figure 7. As can be seen, the average tunnel diameter and the corroded area per unit surface area exhibited a similar trend, both increasing initially and then decreasing with increasing silane concentration. By contrast, the tunnel density decreased continuously. The error bars of the average tunnel diameter and tunnel density are relatively small, indicating that these two parameters show limited spatial variation among different regions of the same sample. By contrast, the corroded area per unit surface exhibits comparatively larger error bars, especially at low silane concentrations, suggesting that this parameter is more sensitive to local tunnel clustering and merged-tunnel formation. This behavior is attributed to the large difference in surface electrochemical activity on the blank aluminum foil during DC tunnel etching, which led to a non-uniform distribution of corrosion current. As a result, a large number of merged tunnels formed in regions with current concentration, whereas only fine etched tunnels were generated in other regions, thereby reducing the average tunnel diameter and the corroded area per unit surface area. Therefore, although the blank aluminum foil exhibited the highest tunnel density, its corroded area per unit surface area remained relatively low. With increasing silane concentration, the etched tunnels became more uniformly distributed, while the tunnel density, average diameter, and corroded area per unit surface area all decreased. This is attributed to the enhanced ability of the silane film to homogenize the corrosion current distribution at higher silane concentrations, resulting in more dispersed pitting initiation sites. Meanwhile, the improved corrosion inhibition effect of the silane film increased the resistance to tunnel initiation, thereby reducing the tunnel density. Since the capacitance performance is closely related to the specific surface area, excessively low tunnel density and average tunnel diameter are unfavorable for maintaining the subsequent capacitor performance. Based on the comparison of the etched tunnel morphologies and structural parameters, the optimal silane electrodeposition concentration was determined to be 3 vol%, at which the etched tunnels exhibited both a relatively high density and a uniform distribution.

**Figure 9:**
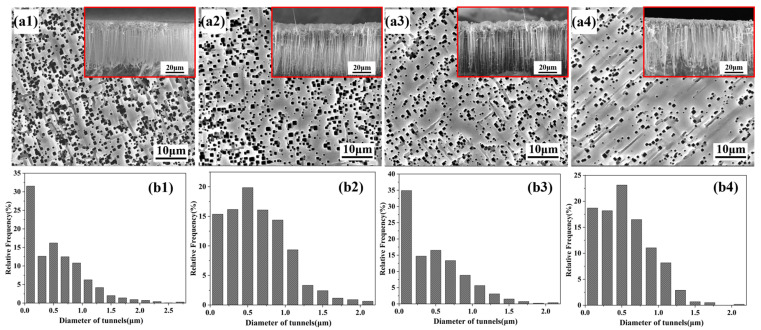
Etched tunnel morphologies and tunnel diameter distributions of aluminum foils at different deposition times: (**a1**,**b1**) 1 min, (**a2**,**b2**) 5 min, (**a3**,**b3**) 10 min, and (**a4**,**b4**) 20 min.

Figure 9 presents the etched tunnel morphologies of aluminum foils after silane electrodeposition at different deposition times. As shown in Figure 9, increasing the electrodeposition time reduced the density of etched tunnels, which can be clearly observed from the cross-sectional morphologies marked by the red boxes in Figure 9(a1–a4), where the etched tunnel density decreases progressively with increasing deposition time. Meanwhile, the uniformity of etched tunnel distribution improved and the number of merged tunnels decreased markedly. This results from the increase in silane film thickness with prolonged electrodeposition time, which enhances its corrosion inhibition effect. A similar trend was also observed in the tunnel diameter distributions. As shown in Figure 9(b1), the etched tunnels on the aluminum foil treated for 1 min exhibited a relatively wide diameter distribution. This reflects the insufficient silane deposition at short treatment time, which leads to a relatively weak corrosion inhibition effect of the silane film. Consequently, etched tunnels tend to cluster and grow in local regions, consuming a large amount of charge and leaving less charge available for tunnel growth in other regions, thereby leading to smaller tunnel diameters in those areas. With prolonged silane electrodeposition, the corrosion inhibition effect on the aluminum foil surface became stronger, and the proportion of etched tunnels with diameters larger than 1.5 μm decreased significantly.

**Figure 10:**
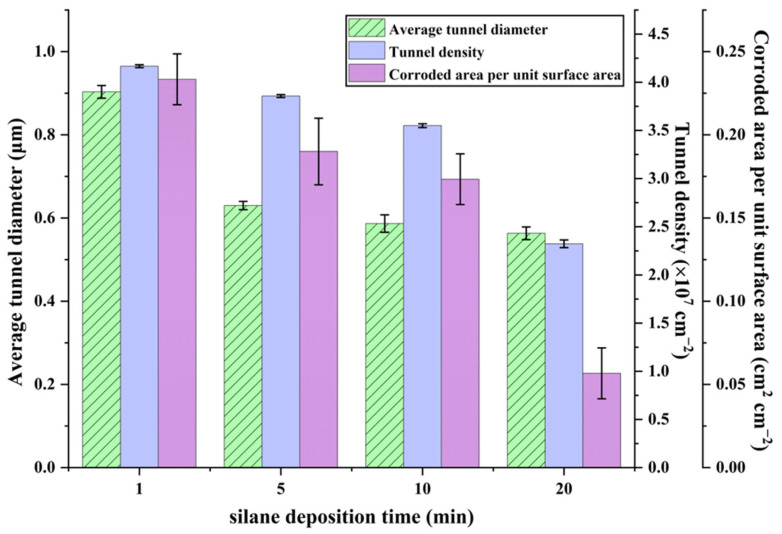
Statistical analysis of the structural parameters of the etched tunnels shown in Figure 9. Error bars represent mean ± SD based on measurements obtained from three SEM images taken from different regions of the same sample (*n* = 3).

Figure 10 presents the statistical results of the etched tunnel structural parameters shown in Figure 9. As shown in Figure 10, the average tunnel diameter decreased markedly with increasing silane electrodeposition time and then remained nearly constant. With increasing electrodeposition time, both the tunnel density and the corroded area per unit surface area decreased slightly at first and then more markedly. The trend of the error bars in Figure 10 is similar to that observed in Figure 8. The average tunnel diameter and tunnel density show relatively small deviations. In contrast, the corroded area per unit surface area exhibits larger fluctuations, particularly at shorter electrodeposition times, reflecting its higher sensitivity to local tunnel coalescence and spatial heterogeneity of the etching process. This is consistent with the evolution of etched tunnel morphology shown in Figure 9. Under short electrodeposition times, the current distribution during DC tunnel etching remained non-uniform, resulting in a relatively large number of merged tunnels. With increasing electrodeposition time, the etched tunnel distribution became more uniform and the number of merged tunnels decreased significantly, leading to a marked reduction in average tunnel diameter. However, when the electrodeposition time became excessively long, the corrosion inhibition effect of the silane film was further enhanced, thereby increasing the resistance to tunnel initiation and resulting in a pronounced decrease in tunnel density. The corroded area per unit surface area was affected by both tunnel density and tunnel diameter; therefore, it showed a correspondingly pronounced change in the ranges where either tunnel density or tunnel diameter varied significantly. Therefore, 5 min was selected as the optimal silane electrodeposition time, at which the etched tunnels exhibited both a relatively uniform distribution and a sufficiently high tunnel density.

**Figure 11:**
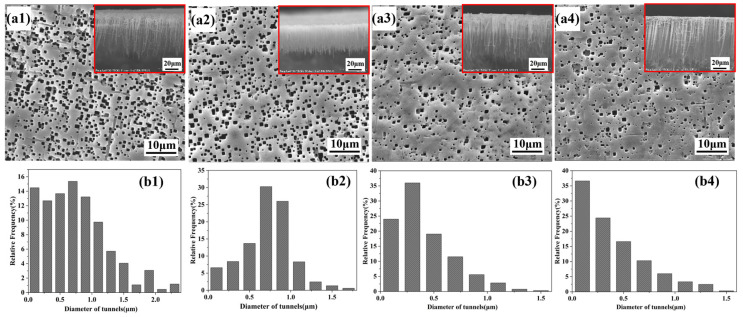
Etched tunnel morphologies and tunnel diameter distributions of aluminum foils at different curing times: (**a1**,**b1**) 0 min, (**a2**,**b2**) 5 min, (**a3**,**b3**) 15 min, and (**a4**,**b4**) 30 min.

Figure 11 presents the etched tunnel morphologies of aluminum foils after thermal curing for different times under the above optimal conditions (3 vol% silane and 5 min electrodeposition time). As shown in Figure 11, thermal curing further reduced the tunnel density, made the tunnel diameter distribution more concentrated, and decreased the maximum tunnel diameter, while the red-boxed cross-sectional morphologies in Figure 11(a1–a4) show the corresponding changes in tunnel length uniformity and tunnel length. This behavior arises from the dehydration condensation between the silane film and the aluminum foil surface during thermal curing, which leads to the formation of stronger Si-O-Al covalent bonds [20,30,31]. This strengthens the interfacial adhesion and further enhances the corrosion inhibition performance of the silane film. As shown in Figure 11(a2,b2), when the curing time was 5 min, the etched tunnels exhibited the best length uniformity, together with a slight decrease in tunnel length. Under this condition, the corrosion current was distributed more uniformly, so that each etched tunnel received a similar amount of charge without an obvious difference in growth sequence. Meanwhile, the relatively high tunnel density led to a lower charge allocation to each individual etched tunnel, thereby resulting in a shorter tunnel length. When the curing time was further increased, the corrosion inhibition effect of the silane film became more pronounced, resulting in a substantial decrease in tunnel density. Consequently, more charge was allocated to each individual tunnel, leading to an increase in tunnel length, as shown in Figure 11(a3,a4).

**Figure 12:**
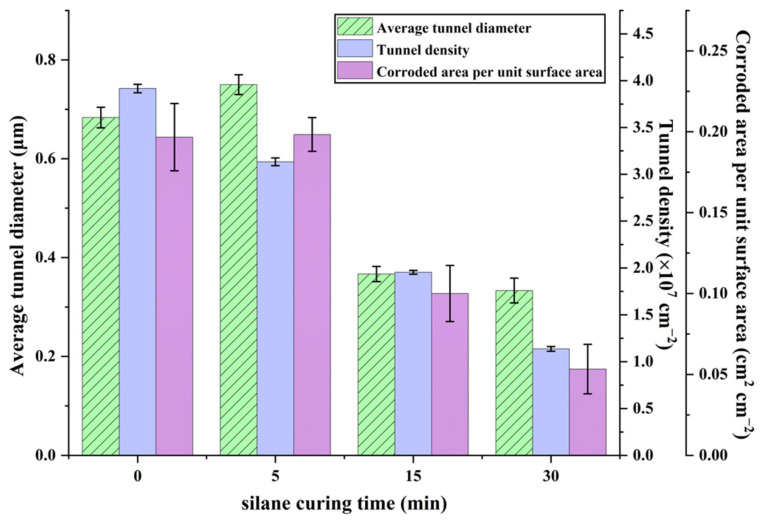
Statistical analysis of the structural parameters of the etched tunnels shown in Figure 11. Error bars represent mean ± SD based on measurements obtained from three SEM images taken from different regions of the same sample (*n* = 3).

Figure 12 presents the statistical results of the structural parameters of the etched tunnels shown in Figure 11. As shown in Figure 12, after thermal curing for 5 min, the tunnel density decreased, whereas both the corroded area per unit surface area and the average tunnel diameter increased. This is because the etched tunnels on the aluminum foil without thermal curing exhibited a relatively broad size distribution, and the presence of a large number of fine tunnels reduced both the average tunnel diameter and the corroded area per unit surface area. After thermal curing, the corrosion inhibition effect of the silane film was enhanced, resulting in a more concentrated tunnel diameter distribution and consequently higher values of both parameters. With further increasing curing time, the tunnel density, tunnel diameter, and corroded area per unit surface area all decreased markedly, which is consistent with the etched tunnel morphologies shown in Figure 11. In addition, the error bars suggest that tunnel density varies only slightly over the whole curing-time range, whereas the corroded area per unit surface area exhibits comparatively larger deviations. The pronounced decreases in tunnel density, tunnel diameter, and corroded area per unit surface area at 15 and 30 min are greater than the regional fluctuations represented by the error bars, indicating that the observed changes in tunnel structure mainly arise from the excessively enhanced corrosion inhibition effect of the silane film rather than from local measurement variability. This further supports the conclusion that 5 min is the optimal curing time, at which a relatively high tunnel density is still maintained while the tunnel distribution is improved. In general, a higher tunnel density, a more uniform tunnel distribution, and an appropriate average tunnel diameter are considered favorable for increasing the effective surface area and, consequently, improving capacitance performance. Under the present conditions, a thermal curing time of 5 min yielded a more desirable tunnel structure, especially with respect to tunnel uniformity and density, suggesting its advantage for capacitance-related performance. As listed in Table 1, the capacitance of the untreated foil was 0.386 μF cm^−2^, while that of the optimally pretreated foil increased to 0.509 μF cm^−2^, representing an enhancement of approximately 31.9%. These results further confirm that the optimized tunnel structure achieved by the combined pretreatment is beneficial for capacitance improvement.

**Figure 13:**
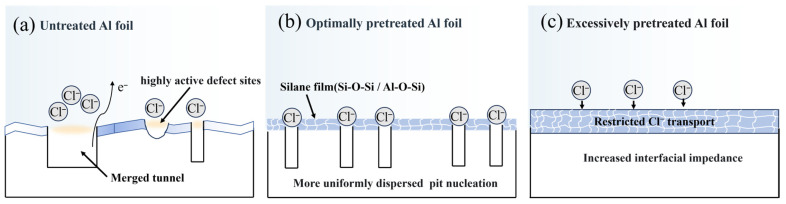
Schematic illustration of the proposed mechanism for tunnel nucleation regulation by combined pretreatment.

Figure 13 schematically illustrates the mechanism by which the combined pretreatment regulates tunnel nucleation on aluminum foil. As shown in Figure 13a, the untreated aluminum foil contains numerous microscopic defects, including rolling marks and cracks in the native oxide film, as a result of rolling manufacturing processes. During the initial stage of DC etching, these defect-rich regions generally possess higher electrochemical activity and are therefore more susceptible to preferential Al dissolution, serving as preferential sites for pit initiation [32,33]. The aggregation of etched tunnels within highly active regions promotes the formation of merged tunnels, thereby reducing the utilization efficiency of the effective surface area. After combined pretreatment, alkaline chemical polishing weakens the rolling-mark-induced surface irregularities and reduces highly active defect regions, as shown in Figure 13b. Meanwhile, BTSE hydrolyzes under mildly acidic conditions to generate silanol groups. During the subsequent thermal curing process, these silanol groups condense with hydroxyl groups on the Al surface to form interfacial Al-O-Si bonds, while self-condensation among silanol groups produces a cross-linked Si-O-Si network [34,35]. The resulting compact silane film moderately increases the interfacial impedance and charge-transfer resistance, partially passivates highly active defect sites, and regulates the transport of Cl^−^ toward the Al surface. Consequently, tunnel nucleation becomes more spatially dispersed, which suppresses tunnel clustering and reduces the formation of merged tunnels [20].

However, when the silane film becomes excessively thick, its role changes from selective regulation to an overly strong barrier effect, as illustrated in Figure 13c. The excessive film strongly restricts Cl^−^ transport and interfacial charge transfer, resulting in a significant decrease in effective nucleation sites and, consequently, a lower tunnel density.

## 4. Conclusions

This work investigated the effects of alkaline chemical polishing parameters and the combined treatment of alkaline chemical polishing and silane electrodeposition on the surface state of aluminum foil and the structure of etched tunnels. The following conclusions can be drawn:

1. The intensity of alkaline chemical polishing increased with increasing NaOH concentration, immersion time, and immersion temperature. Appropriate polishing facilitated the removal of surface rolling marks, improved the uniformity of surface activity, and promoted the subsequent uniform electrodeposition of the silane film. Excessive polishing introduced surface pits on the aluminum foil. These defects acted as preferential sites during the subsequent etching process, promoting localized dissolution and leading to the merging of etched tunnels. The optimal polishing parameters were 4 wt% NaOH, 40 °C, and 3 min.
2. The combined treatment of alkaline chemical polishing and silane electrodeposition improved the uniformity of the etched tunnel distribution and reduced tunnel merging. The optimal silane treatment conditions were 3 vol% silane, 5 min electrodeposition, and 5 min thermal curing. Under these conditions, the etched tunnels exhibited good distribution uniformity and relatively high tunnel density, which was favorable for increasing the effective surface area. Accordingly, the specific capacitance of the etched aluminum foil increased from 0.386 μF cm^−2^ for the untreated foil to 0.509 μF cm^−2^ for the optimally pretreated foil, corresponding to an enhancement of approximately 31.9%.

## Figures and Tables

**Table 1:** Comparison of the specific capacitance of the untreated foil and the optimally pretreated foil.

Sample	Specific Capacitance/μF cm^−2^	Increase/%
Untreated foil	0.386	—
Optimally pretreated foil	0.509	31.9%

## Data Availability

The original contributions presented in this study are included in the article. Further inquiries can be directed to the corresponding author.
